# Demethylating Agents in the Treatment of Cancer

**DOI:** 10.3390/ph3072022

**Published:** 2010-07-02

**Authors:** Paul M. Howell, Zixing Liu, Hung T. Khong

**Affiliations:** University of South Alabama, Mitchell Cancer Institute/1660 Springhill Ave., Mobile, AL 36604, USA; E-Mails: paulhowell@usouthal.edu (P.M.H.); zixingliu@usouthal.edu (Z.L.)

**Keywords:** azacitidine, cancer, decitabine, epigenetics, methylation

## Abstract

Gene silencing resulting from aberrant DNA methylation can lead to tumorigenesis. Therefore, drugs that inhibit or interfere with DNA methylation have been used to reactivate and induce silenced gene re-expression in malignancies. Two demethylating agents, azacitidine and decitabine, are approved for the treatment of myelodysplastic syndromes (MDS) by the U.S. Food and Drug Administration (FDA), and are now considered the standard of care in MDS. In this review, we discuss clinical data, including clinical benefits and toxicities, which led to the approval of azacitidine and decitabine. We also summarize findings from clinical trials that used these two demethylating agents in the treatment of solid tumors. Lastly, we discuss some limitations in the use of azacitidine and decitabine in cancer therapy.

## 1. Introduction

Epigenetics was one of the most popular focuses of cancer research in the last decade. While genetic aberrations such as mutation, deletion and translocation form much of the basis for what we know about acquired and spontaneously developed disease states, including cancer, epigenetics, defined as heritable changes in gene expression that do not involve alterations in DNA sequence, has indeed provided another level of gene expression regulation still present after multiple cell divisions whereby direct DNA modification is not required to promote the development and progression of a tumor. Instead, epigenetic events such as hypermethylation of gene promoter regions (blocking binding sites of transcription factors) [1], global DNA hypomethylation, histone tail modifications (including combinations of methylation and acetylation, among others), small RNA (including microRNA) expression, and higher order chromatin folding all act and interact to manage the expression of individual and collections of genes, responsible for either the maintenance of cellular homeostasis and ‘correct’ activity or, when deregulated, inducing the cell to evade apoptosis, proliferate and potentially invade tissues and metastasize. While many of these processes are not novel, more recent studies tying epigenetic mechanisms to the expression of tumor suppressor genes and oncogenes [1], the subsequent activities of relevant signaling pathways, as well as reporting them as readily detectable independent diagnostic and prognostic tumor biomarkers [2,3,4,5], (McCabe)have propelled this field of research to the forefront of clinical cancer therapeutics. Furthermore, unlike genetic mutations, epigenetic aberrations are inherently reversible, thus making the use of targeted therapies against them very attractive. Over the past decade, various drugs have been developed to target such processes, many with promising results in clinical trials, while some older compounds have become better managed through more calculated dosing regimens to achieve their projected potential as effective antineoplastic agents, namely the hypomethylation-inducing cytidine analogs 5-azacytidine (azacitidine, 5-aza-CR; Vidaza^®^, Celgene Corp., Summit, NJ, USA) and 5-aza-2’-deoxycytidine (decitabine, 5-aza-CdR; Dacogen^®^, SuperGen, Inc., Dublin, CA, USA). 

In this review, we will discuss data from clinical trials using azacitidine and decitabine in hematologic and solid malignancies as well as discuss their therapeutic limitations. In particular, we will focus on the clinical evolution of azacitidine and decitabine and their approval by the U.S. Food and Drug Administration (FDA) for the treatment of myelodysplastic syndromes (MDS).

## 2. DNA Methylation

Of all known mammalian epigenetic modifications, DNA methylation is likely the most widely and intensively studied. As a second tier of gene expression regulation along with chromatin folding, gene promoter methylation provides a physical blockage of the DNA binding site for transcription factors while further inhibiting transcription through the recruitment of chromatin modifying proteins via methyl-CpG binding proteins (MBPs) [6]. Transcriptionally inactive heterochromatin, tightly compacted, typically harbors hypermethylated DNA while active euchromatin is conversely unmethylated [7]. DNA methylation is directed by DNA methyltransferases (DNMT1, DNMT3A, DNMT3B) which transfer a methyl group from S-adenosyl-l-methionine to the cytosine of a CpG (cytosine-phosphate-guanine) dinucleotide (adjacent within a single DNA strand) immediately following replication. Since a CpG on one strand of DNA will pair with a CpG on the other, there exists the possibility for either completely unmethylated (neither CpG), hemimethylated (one CpG) or fully methylated (both CpG:CpG) sites. DNMT1, the most abundant DNMT in mammals, is an important mediator of *de novo* and maintenance methylation of unmethylated and hemimethylated sites, respectively, but preferentially binds hemimethylated sites to restore full CpG pair methylation after replication has resulted in one unmethylated daughter strand [8,9]. When DNMT1 levels are reduced, as is the case following azacitidine or decitabine treatment, daughter strands are less likely to undergo such maintenance to restore full methylation; thus, with each replication, CpG pairs become unmethylated, their promoter regions now more accessible to transcription factors.

In the vertebrate genome, ~37% of CpGs locate in the 5’-regulatory promoter regions of 60–70% of genes in CpG ‘islands’, regions of the genome with a high frequency of CpGs [10,11]. The reason for such a discrepant localization may be explained by CpG developmental character. Methylated CpGs (meCpGs) have an increased propensity for transition mutation to TpG, and since most CpGs are methylated in normal human cells, the relatively high frequency of CpGs in promoter regions has been attributed to their evolutionarily conserved unmethylated status [3], typically maintained during development and differentiation [12]. However, promoter hypermethylation during development is also common and well described [13,14,15], resulting in long-term gene silencing, e.g., X-chromosome inactivation [16] and gene imprinting [17]. 

When methylation machinery becomes deregulated, as through the increased expression/activity of DNMTs, reduced expression/activity of their negative regulators, reactive oxygen species (ROS) formation as due to chronic inflammation, and/or the presence of carcinogens, resulting promoter hypermethylation may lead to a variety of pro-tumorigenic outcomes. For example, when methylated, expression of tumor suppressor genes, such as the retinoblastoma gene (*RB1*), E-cadherin (*CDH1*), and the DNA repair gene O6-methylguanine-DNA methyltransferase (*MGMT*), is reduced, leading to events such as an increased frequency of mutation and microsatellite instability [18,19]. Currently, methylated *MGMT* is being utilized as a valuable marker of chemosensitivity to alkylating agents in patients with gliomas, further emphasizing the translational potential of such epigenetic mechanisms into the realm of cancer prognostics [19,20,21,22]. Of note, CpG islands within the 3’ regions of genes as well as intergenic islands may be hypermethylated in cancer [23,24], while an increased expression of some 3’ meCpG genes has also been described [23]. Such evidence implies for the multifaceted influence of even singular epigenetic processes over gene expression. It is therefore incumbent upon us to continue to study and learn to manipulate such an important epigenetic event for the purpose of inducing silenced tumor suppressor gene expression [25], increasing the possibilities for appropriate apoptotic response and reversing tumor progression.

Differences in methylation patterns of normal and tumor cells were first described around 30 years ago, in particular that tumor cells displayed a global hypomethylation relative to normal cells of the same type [26,27,28,29]. Advancing the early use of Southern blotting and restriction enzymes for methylation profiling, techniques such as bisulfite sequencing with methylation-specific PCR (MSP), array-based detection, and immunoprecipitation of meCpGs have been employed to define cancers based on global DNA methylation status [30]. Indeed, many normal and cancerous tissues have been profiled using these methods, exhibiting commonalities of global hypomethylation and promoter hypermethylation of many recognized tumor suppressor genes [31,32,33] and miRNA [34,35] as well as displaying tissue-specific methylation patterns [31,36]. Furthermore, the interplay of CpG methylation with histone [37,38] and miRNA [35,39,40,41,42] gene regulatory mechanisms makes DNA methylation an important focus of translational cancer research. Given that normal cells typically display unmethylated promoter CpGs, targeted therapeutics against methylation includes an inherent specificity for tumor cells.

## 3. Development and Utility of Azacitidine and Decitabine for Cancer Therapy

### 3.1. Mechanistics

Azacitidine and its deoxy derivative decitabine are cytidine analogs that contain a nitrogen in place of the 5-carbon of the pyrimidine ring. Each enters the cell via nucleoside transporters, such as the confirmed human concentrative nucleoside transporter 1 (hCNT1) and equilibrative nucleoside transporter 1 (hENT1) [43,44,45]. Once inside the cell they undergo three steps of phosphorylation to achieve their active forms. The initial rate-limiting monophosphorylation by uridine-cytidine kinase (UCK; azacitidine) and deoxycytidine kinase (dCK; decitabine) is followed by phosphorylation to their diphosphate forms. Reduction of ~10% of azacitidine diphosphate to its deoxy form, decitabine diphosphate, by ribonucleotide reductase allows both drugs to target DNA, albeit with different potencies, as decitabine triphosphate can incorporate into DNA inducing CpG demethylation and chromosomal instability, while the remaining ~90% of azacitidine triphosphate incorporates into messenger and transfer RNA, inhibiting protein synthesis. Importantly, since decitabine phosphorylation only proceeds toward targeting DNA, it exhibits a more potent inhibition of DNA methylation and is at least 10 times more cytotoxic than azacitidine [46,47,48]. Cytidine deaminase acts to deaminate and open up the azanucleotide ring structure leading to metabolism of the nucleotide [49] with primary excretion via the kidneys; however, van Groeningen *et al.* reported on the significant role of metabolic breakdown of decitabine in the body as they observed high drug clearance with less than 1% of the administered dose excreted in urine in a phase I trial of 21 patients with advanced solid tumors [50].

DNMT1 irreversibly binds DNA-incorporated decitabine triphosphate, reducing available DNMT1 in the nucleus through sequestration and its subsequent degradation, thus passively inhibiting DNA methylation [46,51,52,53]. Clinically, DNMT1 protein levels may be assayed for to assess incorporation of the azanucleoside [54]. Furthermore, the binding of DNMT1, typically found in multi-unit enzyme complexes with chromatin remodelers such as histone deacetylases (HDAC) [55,56], to an azanucleotide may induce structural and functional changes in the complex, reducing chromatin folding and further promoting gene expression. 

The pharmacologic effects of high-dose azanucleosides may be due in large part to the cytotoxicity of azanucleotide:DNMT adducts formed in DNA, potentially masking the importance of demethylation via clonal expansion of resistant cells [46,57]. Lower doses instead induce adduct degradation without inhibition of DNA synthesis, thus allowing for optimal extended treatment durations [58]. As demethylation induces the re-expression of silenced genes from various pathways, e.g. apoptosis, differentiation, angiogenesis, senescence, *etc.*, many of which have tumor and patient-specific activity, we must also keep in mind the inherent difficulties associated with such non-specific targeting with demethylating agents in predicting patient response.

### 3.2. Development and Preliminary Data

Piskala and Sorm first described their synthesized nucleoside analogs azacitidine and decitabine as highly active cytotoxic agents against lymphatic leukemia in mice and cell lines, able to induce chromosomal breakage [46,59], nearly 45 years ago [60,61]. Each was further shown to induce differentiation of multipotent mouse embryonic 10T1/2 cells [62,63], later reported to be the result of their inhibition of DNA methylation [64,65]. Hematopoietic cancers have a particularly high degree of aberrant methylation [66]. Further supporting the roles of azanucleosides in differentiation, an increase of fetal hemoglobin was observed after treatment with azacitidine in patients with β-thalassemia [67] and sickle cell anemia [68], and monoblastic and myeloblastic leukemia cells were induced to differentiate upon treatment with decitabine [69]. Multiple treatment regimens of decitabine in L1210 leukemic mouse xeonografts showed promising increases in life span with a significant lag in tumor cell proliferation [70], and greater than 70% inhibition of DNA methylation was observed in blood cells from patients with lymphatic and myeloid leukemias [48]. Early data from *in vivo* solid tumor models showed little effect [61]; however, with the continuous identification of methylated tumor suppressors in solid tumors came a heightened interest in optimizing drug delivery systems and dosing and reducing toxicity for future therapy. 

1971 saw an application by the National Cancer Institutes to the FDA for Investigational New Drug status, indicating azacitidine as an antineoplastic agent for the treatment of multiple cancers. Clinical trials had begun in Europe in 1967 and in the U.S. in 1970 including more than 800 evaluable patients with such conditions as acute and chronic myelogenous leukemia (AML, CML), acute lymphocytic leukemia (ALL), and breast, colorectal, lung, and melanoma solid tumors [61]. Azacitidine was quite effective in treating AML, in particular for patients with an initial or developed resistance to common treatment regimens of that time and/or with relapse, thus providing a valuable niche for azacitidine. Of 200 patients, 41 (20%) exhibited complete disease remission with 32 (16%) partial remissions following treatment with azacitidine, the majority of responding patients having been refractory to conventional chemotherapeutics. Administration in these studies ranged from 15 doses of 60 mg/m^2^ every eight hours to five daily doses of 500 mg/m^2^ to a single infusion of 750 mg/m^2^. However, responses were either low or non-existent in CML, ALL and multiple myeloma. Data from solid tumor trials either showed poor response or were inadequate for deriving any significant conclusion. Toxicity was particularly disconcerting as 73% of all 745 patients reported to the Investigational Drug Branch exhibited nausea and vomiting within three hours of each series of intravenous (i.v.) injections, and 53% of patients exhibited diarrhea. Sporadic yet occasionally severe myalgia was reported while incidences of dose-limiting leukopenia and thrombocytopenia were 34% and 17%, respectively. Overall, few deaths were associated with treatment, most (N = 4) reported in patients with unhealthy livers [71]. Importantly, an escalation toward maximum tolerated dose, a common protocol for early trials, may not be the best way to observe such a drug, as azacitidine is most efficacious at low doses, inhibiting DNA synthesis at high doses [58,61,72]. A more relaxed regimen of 75 mg/m^2^d for seven days every 28 days would later prove more effective and less toxic in a subset of cancer patients.

The FDA rejected this application for azacitidine due at least in part to an unacceptable level of toxicity relative to observed antitumor effectiveness; however, these collective data served to promote intrigue regarding the actions and development of epigenetic therapies for cancer. Four decades would ultimately pass before the first such drug gained approval for clinical use, carrying with it an immediate impact in the treatment of hematologic malignancies, in particular MDS.

### 3.3. FDA Approval for Myelodysplastic Syndromes

#### 3.3.1. Myelodysplastic Syndromes

MDS are a collection of hematopoietic stem cell disorders characterized by bone marrow dysplasia and peripheral blood cytopenia, sometimes referred to as preleukemias due to their tendency to transform into AML [73]. Five subtypes of MDS are designated under the original French-American-British (FAB) classification system, including: (1) refractory anemia (RA); RAs with (2) ring sideroblasts (RARS; high iron content in red blood cells); (3) excess blasts in bone marrow (RAEB) and a (4) high excess of blasts in transformation to AML (RAEB-T; low level of circulating white blood cells and platelets); as well as (5) chronic myelomonocytic leukemia (CMML; high level of circulating monocytes, variable red and white blood cells and platelet levels) [74]. The World Health Organization (WHO), the International Working Group (IWG), sponsored by the National Cancer Institutes (NCI), and International Prognostic Scoring System (IPSS), the most common prognostic scoring system for MDS, have since made important adjustments to the somewhat dynamic classification and response criteria of MDS, with the notable exclusion of CMML [75,76,77,78,79]. 

The cellular heterogeneity of MDS provides a valuable prognostic tool for a historically difficult cancer to treat. Bone marrow mononuclear cells from MDS patients exhibited gene expression signatures similar to AML, MDS and non-leukemic cells in a 1:2:1 relationship, respectively, while AML-like cells were in abundance (68%) in WHO-designated RAEB-2 (high-risk, transforming) marrow [80]. Most high-risk, RAEB malignancies result in patient mortality within one year, nearly half transforming into AML [81]. Unfortunately, effective treatment of these patients has been problematic in the past as high-dose cytotoxic agents have produced generally poor results. Importantly, as most patients diagnosed with an MDS are over 70 years old [82], allogeneic hematopoietic stem-cell transplantation (SCT), which produces the best long-term disease remission but also has a high rate of treatment-related death (39% after one year) and significant graft-*versus*-host disease [83], is not warranted except in cases of advanced disease [84], encompassing only about 5% of all MDS patients [85]. Supportive care, sometimes including blood transfusion, administration of hematopoietic growth factors, or chemotherapy, had generally been the standard of care for many low- and high-risk MDS patients prior to the availability of azacitidine and decitabine with effective dosing regimens. Notably, recent studies indicate the feasibility of pre-treating high-risk MDS/AML patients with decitabine prior to SCT, citing no unexpected toxicities [86,87]. 

#### 3.3.2. Azacitidine

In a second Investigational New Drug application, this time for the specific use of azacitidine in patients with MDS, Pharmion Corp. (acquired by Celgene Corp. in 2008) submitted data from three open-label, multicenter trials of the Cancer and Leukemia Group B (CALGB) [81,85,88,89]. After initial data was generated under CALGB and original FAB criteria, a reanalysis using updated WHO and IWG classification and response criteria, respectively, was completed [90]. CALGB 9221 [85] was the lone controlled trial testing subcutaneous (s.c.) injections of azacitidine in patients with all five types of MDS against those solely under supportive care, while CALGB 8921 [88] and 8421 [89] tested s.c. and i.v. injections, respectively, without a control arm. Marcucci *et al*. have since reported a two-fold higher beta (substance elimination) half-life for s.c. injections of azacitidine (41 min) over i.v. (20 min) in patients with MDS [91]. Dosing for all trials was 75 mg/m^2^d given for seven consecutive days every 28 days. All staging and response criteria were identical for the three trials. Patients with an adjudicated AML diagnosis at baseline were not included in the analyses of transformation to AML and response rate. Complete response (CR) is defined as complete normalization of bone marrow and peripheral blood counts; partial response (PR) as > or = 50% restoration of the deficit from normal of all three peripheral blood cell lines, elimination of transfusion requirements, and a decrease in percentage bone marrow blasts by > or = 50% from pre-study values; hematologic improvement (HI) as > or = 50% restoration in the deficit from normal of one or more peripheral blood cell lines and/or a > or = 50% decrease in transfusion requirements; and total measurable response as CR + PR + HI.

An early CALGB phase II trial [89] included 43 evaluable hospitalized patients with high-risk RAEB or RAEB-T. Under original CALGB/FAB criteria, complete response was seen in five (12%) patients, partial response in 11 (25%), and hematologic improvement in five (12%) patients for a total measurable response in 21 (49%) patients. Median survival for all patients was 13.3 months with median duration of response for those with complete and partial response at 14.7 months. Median time to response was three cycles. Toxicities included nausea and vomiting (63% incidence) and dose-limiting myelosuppression (33%).

The second phase II trial, CALGB 8921 [88,92], exhibited similar response rates, duration and survival to the first, albeit via an s.c. daily bolus injection of an evaluable 70 patients of RAEB, RAEB-T and CMMT subtypes. Complete responses were noted for 12 (17%) patients and hematologic improvement for 16 (23%), while toxicity, save for an occasional skin reaction at site of injection, and median time to response were similar to the other trials.

In the multicenter (26 academic centers, 30 affiliates), randomized, open-label and controlled CALGB 9221 trial [85,92], 191 patients encompassing each MDS subtype, including 20 AML, were stratified according to FAB classification and randomly separated into azacitidine treatment arm (N = 99) and observational, supportive therapy arm (N = 92), additionally matched across arms according to patient physical (e.g., age, gender, race, weight), disease (e.g., FAB subtype, cytogenic analysis, IPSS classification, time from diagnosis to study entry), and treatment history characteristics. Patients were allowed to cross over from the observational to treatment arm after a minimum of four months if disease progressed, whereby they were treated and studied identically to those initially randomized to the treatment arm. Four cycles after azacitidine treatment, patient bone marrow biopsies were analyzed. Patients with complete response continued on treatment for an additional three cycles, while those with partial response or hematologic improvement continued until achieving a complete response or relapse. 

Of 99 patients in the treatment arm, seven (7%) exhibited CR; 16 (16%), PR; and 37 (37%), HI, for a total measurable response of 60 (60%), each a statistically significant response as compared to observation. Alternatively, only five of 92 (5%) patients in the supportive care arm showed any response (HI). Median time to initial response and best response was 64 days (cycle 3) and 93 days (cycle 4), respectively, as median duration of response for those with CR, PR or HI was 15 months. Patients who either crossed over from the observational to the treatment arm after six months or did not cross over at all exhibited a significantly lower median overall survival (11 months) than those initially randomized to the azacitidine arm (18 months). Furthermore, of the 49 who did in fact cross over at some point, 23 (47%) responded to treatment with five (10%) patients achieving CR; two (4%), PR; and 16 (33%), HI. Median overall survival was 20 months for all patients in the azacitidine arm *versus* 14 months for those who remained under supportive care. Time to progression to either AML or death was significantly (P = 0.04) longer (19 months) than for that of the observational arm (eight months). During the first six months of the trial, 3% of patients in the azacitidine arm transformed into AML, while 24% from observation transformed (P < 0.0001). 

Quality of life was also shown to improve significantly for those treated with azacitidine, including those who crossed over [93], compared with the observational arm, which either remained stable or worsened with time. Toxicities associated with azacitidine treatment include transient cytopenias, grade 3 or 4 leukopenia (43% incidence), granulocytopenia (58%), and thrombocytopenia (52%) with bleeding as well as infection (20%), nausea and vomiting (4%), and one potentially treatment-related death. Other common events include myalgia, weakness, fatigue, rash, erythema, limb pain, neutropenia, pneumonia, coughing, dyspnea, constipation, and fever. The frequency of such events decreased after the first two cycles of therapy. Fifty patients were discontinued from the trials for showing no response after four cycles of treatment. Overall, azacitidine is a significantly more effective antineoplastic agent than supportive care for MDS, improving overall survival, bone marrow function and quality of life while reducing and delaying MDS transformation into AML.

The application of updated WHO, IWG and IPSS criteria to data from the CALGB trials has served to validate these findings, the major change being a more precise definition of RAEB and RAEB-T, evident by the increased number of RAEB and RAEB-T patients re-characterized as AML in each trial [90]. Overall response rates indeed remained consistent. On May 19, 2004, the FDA approved azacitidine (Vidaza^®^) as an injectable suspension for the treatment of all five FAB subtypes of MDS (including RARS, if accompanied by neutropenia or thrombocytopenia or requiring transfusions) [92]. 

#### 3.3.3. Decitabine

The efficacy of decitabine infusion in pediatric and adult leukemia patients has been described, resulting in 20% and 33% complete response rates for those with ALL and AML, respectively [94]. Phase II trials of decitabine (45 mg/m^2^d for three days every six weeks) as treatment for MDS in older patients resulted in complete response rates of 20-28% and overall response rates of 42–54% [95,96]. 

These data led to the initiation of a multicenter, open-label, randomized, and controlled phase III trial for decitabine *versus* supportive care in 170 patients with all five FAB sub-types of MDS and IPSS-designated intermediate-1, intermediate-2 and high-risk, 70% of all patients being of intermediate-2 or high-risk IPSS type [97]. Patients were randomized into two groups, decitabine plus best supportive care (N = 89) and solely best supportive care (N = 82). The dosing regimen was continuous i.v. infusion of 15 mg/m^2^ over three hours every eight hours for three days, repeating every six weeks.

Durable response seen for patients in the decitabine arm was 17% (15 of 89 patients, 9% CR + 8% PR) with 13% HI, and 0% in the control, supportive care arm (P<0.001). For the decitabine arm, median time to response was 3.3 months and median duration of response was 10.3 months. Patients in the decitabine arm also tended to have a longer median time to develop AML or death than patients in the supportive care group (all patients, 12.1 *vs.* 7.8 months, P = 0.16), and statistically significant values were seen for intermediate-2/high-risk (P = 0.03) and *de novo* (P = 0.04) disease groups. Two multicenter, open-label, single-arm trials of decitabine (15 mg/m^2^ continuous infusion for four hours every eight hours over three days, repeating every six weeks) for 164 total patients with MDS of any FAB sub-type were further initiated, resulting in overall response rates of 26% (N = 66) and 24% (N = 98) [98]. Nausea, vomiting, constipation, diarrhea, fever, hyperglycemia, back pain, coughing, headache, insomnia, rash, and petechiae were among the more common side effects, while the primary toxicity of decitabine was myelosuppression.

On May 2, 2006, the FDA approved decitabine (Dacogen^®^) as an injectable suspension for the treatment of all original FAB subtypes of MDS as well as intermediate-1, intermediate-2, and high-risk groups of the IPSS. Recently, March 11, 2010 saw the FDA approval of a five-day out-patient dosing regimen for Dacogen^®^ for injection. The regimen consists of a 20 mg/m^2^ continuous i.v. infusion over one hour repeated daily over a five day cycle, repeating every four weeks. This regimen aims to improve upon the previous in-patient dosing of 15 mg/m^2^ i.v. over three hours repeated every eight hours for three days per cycle, repeating every six weeks. Three multicenter, open-label, single-arm studies evaluated the efficacy of this decitabine treatment regimen for patients with MDS of any FAB sub-type [99]. Based on IWG 2000 criteria, the overall response rate was 16% (15% CR, 1% PR) with a median time to response of 162 days and median duration of response of 443 days. Hematologic toxicities, including neutropenia (37%), thrombocytopenia (24%) and anemia (22%), and infections were the most prevalent toxicities, accounting for most dose delays and patient discontinuation, possibly contributing at least in part to eight infection- and/or bleeding-associated deaths. Fatigue, nausea, coughing, constipation, and diarrhea were listed among common adverse events.

### 3.4. Azacitidine, Decitabine and Chemotherapy for High-Risk MDS Patients

Current National Comprehensive Cancer Network guidelines recommend the use of hypomethylating agents for IPSS-classified high-risk MDS patients who are not candidates for conventional chemotherapy.

In a recent multicenter, randomized trial for patients of all prognostic sub-groups of high-risk MDS, azacitidine (75 mg/m^2^d for seven days every 28 days) nearly doubled their overall survival rate (50.8% *vs.* 26.2%) past that of conventional therapy, including best supportive care, low-dose cytarabine or intensive chemotherapy [100]. The study, carried out by the International Vidaza^®^ High-Risk MDS Survival Study Group, enrolled 358 patients at 79 sites in 15 countries. Azacitidine induced more serious hematologic side effects than the best supportive care, but fewer than chemotherapy, and it lowered the risk of patient infection by one-third compared with conventional care. However, there was no significant advantage over chemotherapy, potentially due to the few available patients suitable for chemotherapy. Response rates of 17% complete and 12% partial indicated little benefit over chemotherapy or combination treatment [101]. Data from a meta-analysis of three trials including over 900 MDS patients testing azacitidine and decitabine against these same conventional therapies suggests instead a prolonged overall survival and time to AML transformation or death, improved CR, PR, HI, and overall response after treatment [102]. A phase III clinical trial has recently been initiated to demonstrate the “superiority” of decitabine over azacitidine for the treatment of these intermediate- or high-risk MDS patients (http://www.clinicaltrials.gov/, NCT01011283).

## 4. Azacitidine and Decitabine in the Treatment of Solid Tumors

Demethylating agents seem to be more effective in patients with hematologic malignancies rather than solid tumors and effective at much lower doses; however, mountains of basic research data tell us that methylation is a common driving force behind many facets of tumorigenesis, growth and metastasis [103]. Indeed, a PubMed search for “DNA methylation cancer” returns more than 12,000 articles. While we can efficiently utilize azanucleosides for the manipulation of numerous types of solid tumor cells *in vitro*, their translation to therapeutics has been somewhat of a mystery. With all that we now know about epigenetic regulation of cancer progression via DNA methylation, effective treatment of solid malignancies with demethylating agents in the clinical setting seems to be just around the corner.

The use of both azacitidine and decitabine in clinical trials for solid tumors has been and is now only occasionally attempted, though the main focus of these drugs for years has undoubtedly regarded hematological malignancies. Solid tumor trials have included gastrointestinal, lung, ovarian, prostate, breast, and head and neck cancers, melanoma and malignant mesothelioma [104,105,106,107,108,109,110,111,112,113,114,115]. While some groups have shown strong response (seven of 11 breast cancer patients (63%) responded to i.v. administration of 300–700 mg/m^2^ over an eight day period), others with larger patient cohorts give little to no response at all. Given the recent success of the azanucleosides in treating MDS, AML and other hematologic cancers, further study into the *in vivo* action of demethylating agents in solid tumor systems is highly warranted.

In a small clinical trial, Momparler *et al.* treated 15 patients with stage IV non-small-cell lung carcinoma with a decitabine regimen of 200–600 mg/m^2^ over eight hours [109]. Median survival for nine evaluable patients was 6.7 months, with three patients surviving at least 15 months. Hellebrekers *et al.* have described the *in vitro* and *in vivo* anti-angiostatic abilities of decitabine, potentially adding another important mechanism for its anti-neoplastic activity [116]. A 168 hours continuous i.v. infusion of decitabine in 10 patients with refractory solid tumor has been described as well tolerated [117]. Promoter-specific and global DNA methylation levels were significantly decreased by day 14, expression change verified by quantitative RT-PCR, with reversion back to baseline 28 to 35 days after treatment had commenced, indicating the transient effect of decitabine on *in vivo* methylation. In 1983, a phase I trial and pharmacokinetic study of decitabine in 21 patients with advanced solid tumors resulted in one partial response [50]. Nineteen patients with metastatic solid tumors were given a dose between 20 and 40 mg/m^2^ via continuous i.v. infusion for 72 hours. After seven days, some gene demethylation was identified, but there were no objective responses to the treatment [118]. 

Beyrouthy *et al.* describe the hyper-sensitization of pluripotent embroyonal carcinoma (EC) cells to low levels (IC_50_, 5–25 nmol/L) of decitabine in the presence of DNMT3B [119]. Additionally, cisplatin-resistant EC cells, the ‘stem cells’ of testicular germ cell tumors, may be re-sensitized to cisplatin toxicity after pre-treatment with decitabine in the presence of DNMT3B. 

Another recent study found that azacitidine therapy significantly reduced median prostate-specific antigen (PSA) doubling time, a sign of improved long-term patient outcome, in men with chemonaïve castration-resistant prostate cancer, correlating with decreased plasma DNA long interspersed nuclear element 1 (LINE-1) methylation levels [120]. The older majority of prostate cancer patients may benefit from such therapy as only minor toxicities were reported.

We have recently initiated a single-center phase I/II clinical trial testing combination azacitidine with the chemotherapeutic, nanoparticle albumin-bound paclitaxel (nab-paclitaxel; Abraxane^®^, Abraxis Bioscience, Los Angeles, CA), for the treatment of patients with advanced or metastatic solid tumors and breast cancer is currently recruiting participants (http://www.clinicaltrials.gov/, NCT00748553). The effective antineoplastic ability of nab-paclitaxel within some tumors is thought to result from its accumulation at the tumor site via the actions of SPARC, secreted protein acidic and rich in cysteine, a secreted glycoprotein overexpressed in a variety of tumors [121]. The presence of bound albumin allows for receptor-mediated transcytosis of nab-paclitaxel across the endothelium. Upon entering the tumor interstitium, SPARC then binds and sequesters albumin, releasing paclitaxel inside the tumor. Low SPARC expression has been described in colon, lung, ovarian, pancreatic, and cervical cancer cell lines, correlating with promoter hypermethylation in tested cases [122,123,124,125,126,127]. Use of a demethylating agent has been shown to induce SPARC expression in some of these cell types [123,126]. In a recent study, SPARC expression was able to inhibit breast cancer metastasis [128]. Our clinical trial intends to up-regulate SPARC with an initial treatment of azacitidine to increase the efficacy of nab-paclitaxel and joint effectiveness of both drugs in solid tumors.

## 5. Limitations of Azacitidine and Decitabine

An important limitation for such demethylating agents is their inherent need for actively dividing cells (S phase) in which to incorporate. Given the short half-life of azacytosines in the body, slow-growing tumors may require a longer dosing schedule, thereby increasing the possibility for treatment-related toxicity. One mode of circumventing this problem is through better drug delivery. For example, chemically modifying azanucleosides to improve their plasma stability and reduce drug degradation may allow the administration of lower doses over longer periods of time. To this end, decitabine can be contained within the dinucleotide S110, imparting it with a greater degree of resistance to the inactivating effects of deamination, while exhibiting comparable abilities for demethylation and inducing tumor cell growth inhibition as decitabine alone [129].

The inherent lack of specificity of azacitidine and decitabine for target genes allows for certain undesirable effects, e.g., global demethylation by azacitidine and decitabine may result in the expression of oncogenic loci and activation of transposable elements [130,131]. Decitabine has indeed been shown to induce the expression of *MDR1*, a gene implicated in drug resistance [132]. Furthermore, resistance to the cytotoxic effects of azacytosines has been noted regardless of degree or stability of incorporation into DNA, for various treatment regimens, and correlated with decreases in both drug-induced hypomethylation of long interspersed nuclear elements (LINEs) and levels of hENT1 [43,44,46,47,133,134]. Likewise, decreases in dCK and increases in cytidine deaminase may confer drug resistance [44,47,135,136]. 

## 6. Current Research Trends

The advent of epigenetic research of modified genes, regions of chromatin, histones, miRNA, and other modified/deregulated proteins in cancer has led to the discovery of many novel and useful methylation biomarkers. Notably, decreased expression via hypermethylation of the *p15^CDKN2B^* cyclin-dependent kinase tumor suppressor gene is recognized in multiple cancers [137] including MDS, where its identification in early MDS is a marker of poor survival and transformation to AML [138]. P15 expression was increased in nine of 12 patients with hypermethylated *p15* upon treatment with decitabine, correlating with hematologic disease reversion including 3 complete responses [139]. Additionally, detection of methylated DNA in patient serum is minimally invasive and may be beneficial for novel biomarker identification and patient tumor characterization for tailoring therapy. Indeed, LINE-1 methylation levels as detected in plasma DNA may be a prognostic marker for chemosensitivity in patients with solid tumors [140].

MicroRNAs are a set of small (~22 nt) non-coding RNAs that bind the 3’-UTR of mRNAs and block translation. They are quickly becoming recognized as vital gene expression mediators during normal biological processes and disease states, including cancer. Blum *et al.* have reported on the response-predictive ability of *miR-29b* in older AML patients treated with decitabine [141]. A phase II clinical trial of single-agent decitabine in 53 older subjects (median 74 years; range, 60-85) with previously untreated CML resulted in an overall response rate of 64% (complete remission, 47%; incomplete remission, 17%) with overall survival for all subjects at 55 weeks (median disease-free survival those with CR, 46 weeks). Cycles of 20 mg/m^2^ were variable and tailored to patient response and toxicities, responding patients typically having received one to two 10 day cycles (leading to incomplete CR) followed by one to two 4-5 day cycles (full CR). *MicroRNA-29b* (*miR-29b*) RNA levels as well as its mRNA targets, the DNA methyltransferases (*DNMT1*, *DNMT3A* and *DNMT3B*), were assayed for in 23 patients with available pre-treatment samples. *miR-29b* levels were significantly higher (P = 0.02) in responders (CR + incomplete CR; N = 14) than in non-responders (N = 9), while *DNMT3A*, the only target with differing expression, tended to be lower (P = 0.06) in responders than in non-responders. As DNMTs are important regulators of gene expression and protein function via gene promoter and protein methylation, respectively, in normal and cancer cells, microRNA thus represent an important upstream mediator of cancer development, progression and chemosensitivity.

Methylation frequency in a tumor sample may additionally be of diagnostic and prognostic value in multiple cancers. Indeed, Shen *et al.* have reported on the predictive value of a methylation profile of 10 genes in MDS patients, citing shorter median overall survival (12.3 *vs.* 17.5 months: P = 0.04) and progression-free survival (6.4 *vs.* 14.9 months; P = 0.009) in patients with greater methylation of these genes than in those with lower methylation (N = 89), further validated in two large patient cohorts (N = 228) [142]. Another recent publication relates the progression of MDS to AML to changing methylation patterns [143]. CpG methylation was more frequent and widespread than chromosomal aberrations in each of 184 MDS and AML patient bone marrow samples, meCpG frequency doubling from 6% of CpG loci in early, low-risk MDS to 12% after transformation to RAEB or AML. Furthermore, significantly more genes were methylated in RAEB/AML samples than in MDS or controls, including the independent prognostic marker frizzled-9 (*FZD9*), a Wnt/beta-catenin signaling receptor predictive of decreased survival in MDS/AML patients when methylated.

Novel uses for epigenetic drugs are also being identified. Radiosensitization using combinations of DNMT and HDAC inhibitors has been proposed based on the tumor-specific sensitization to therapeutics imparted by epigenetic drugs via induced tumor suppressor gene expression in epigenetically repressed chromatin, an uncommon occurrence in normal cells [144]. Administration of oral decitabine at doses 17–34 times the optimal s.c. dose has been shown to reactivate fetal hemoglobin, demethylate the epsilon- and gamma-globin gene promoters, and increase histone acetylation of these promoters in baboons [145]. A recent publication by Garcia-Manero *et al.* reports on the feasibility for future cancer therapy of an oral azacitidine coated in a film to reduce its rapid breakdown in the body, thus increasing its bioavailability [146]. Celgene Corp. has recently included its own oral azacitidine in two phase I multicenter, open-label dose escalation trials for patients with MDS, CMML, AML, lymphoma, and multiple myeloma, assessing individual pharmacokinetics and pharmacodynamics alongside that of parenteral Vidaza^®^ (www.clinicaltrials.gov, NCT00528983, NCT00761722).

## 7. Conclusions

The DNA methyltransferase inhibitors, azacitidine and decitabine, are two of a growing number of drugs designed to target epigenetic processes commonly deregulated during the development and progression of cancer. This class of compound has become a major contributor to basic and translational cancer research, easily one of the most valuable tools available for examining biological trends and implications of DNA methylation in normal and tumorigenic tissues. While they tend to exhibit a greater ability as solo agents to treat hematological malignancies than solid tumors, many groups are finding improvement in a multitude of cancers when combined with other agents, in particular with HDAC inhibitors, or when using different dosing schedules or modes of administration. As effective low doses allow for azacitidine and decitabine to be generally well tolerated, demethylating agents as a whole will continue to be utilized as and influence novel therapeutic interventions for cancer patients.

## Supplementary Files

Supplementary File 1 Correction (PDF, 19 KB)
