# Supplementary material for: Demethylating Agents in the Treatment of Cancer
Source: Pharmaceuticals (Basel). 2010 Jul 2;3(7):2022–44. doi: 10.3390/ph3072022 (PMC4036667; doi:10.3390/ph3072022)
Supplement: Supplementary File 1 — Correction (PDF, 19 KB) [file pharmaceuticals-03-02022-s001.pdf]

Pharmaceuticals **2010**, *3*

OPEN ACCESS

pharmaceuticals

ISSN 1424-8247

www.mdpi.com/journal/pharmaceuticals

Correction

Howell, Jr., P.M. *et al*. Demethylating Agents in the Treatment of Cancer. *Pharmaceuticals* 2010, *3*, 2022-2044

Paul M. Howell, Jr. , Zixing Liu and Hung T. Khong *

University of South Alabama, Mitchell Cancer Institute/1660 Springhill Ave., Mobile, AL 36604, USA; E-Mail: paulhowell@usouthal.edu (P.M.H.); zixingliu@usouthal.edu (Z.L.)

* Author to whom correspondence should be addressed; E-Mail: hkhong@usouthal.edu (H.K.); Tel.: +1-251-445-6000; Fax: +1-251-460-6994

Received: 15 October 2010 / Published: 18 October 2010

We found following errors in our paper recently published in Pharmaceuticals [1]. On page 2038, there are two references [60]. The second one should be reference [61]. Additionally, the following three statements on page 2026 should be adjusted replacing reference [61] with [59] (Von Hoff):

a) Early data from *in vivo* solid tumor models showed little effect [61].

b) Clinical trials had begun in Europe in 1967 and in the U.S. in 1970 including more than 800 evaluable patients with such conditions as acute and chronic myelogenous leukemia (AML, CML), acute lymphocytic leukemia (ALL), and breast, colorectal, lung, and melanoma solid tumors [61].

c) Importantly, an escalation toward maximum tolerated dose, a common protocol for early trials, may not be the best way to observe such a drug, as azacitidine is most efficacious at low doses, inhibiting DNA synthesis at high doses [58,61,72].

References

1. Howell, P.M., Jr.; Liu, Z.; Khong, H.T. Demethylating Agents in the Treatment of Cancer. *Pharmaceuticals* **2010**, *3*, 2022-2044.

© 2010 by the authors; licensee MDPI, Basel, Switzerland. This article is an open access article distributed under the terms and conditions of the Creative Commons Attribution license (http://creativecommons.org/licenses/by/3.0/).
